# Measuring the Moment-to-Moment Variability of Tinnitus: The TrackYourTinnitus Smart Phone App

**DOI:** 10.3389/fnagi.2016.00294

**Published:** 2016-12-15

**Authors:** Winfried Schlee, Rüdiger C. Pryss, Thomas Probst, Johannes Schobel, Alexander Bachmeier, Manfred Reichert, Berthold Langguth

**Affiliations:** ^1^Department of Psychiatry and Psychotherapy, University of RegensburgRegensburg, Germany; ^2^Institute of Databases and Information Systems, Ulm UniversityUlm, Germany; ^3^Department of Psychology, University of RegensburgRegensburg, Germany; ^4^Department of Psychology and Psychotherapy, University of Witten/HerdeckeWitten, Germany

**Keywords:** chronic tinnitus, smartphone application, ecological momentary assessment, moment-to-moment analysis, crowd sourcing, ecological validity

## Abstract

Tinnitus, the phantom perception of sound without a corresponding external sound, is a frequent disorder which causes significant morbidity. So far there is no treatment available that reliably reduces the tinnitus perception. The research is hampered by the large heterogeneity of tinnitus and the fact that the tinnitus perception fluctuates over time. It is therefore necessary to develop tools for measuring fluctuations of tinnitus perception over time and for analyzing data on single subject basis. However, this type of longitudinal measurement is difficult to perform using the traditional research methods such as paper-and-pencil questionnaires or clinical interviews. Ecological momentary assessment (EMA) represents a research concept that allows the assessment of subjective measurements under real-life conditions using portable electronic devices and thereby enables the researcher to collect longitudinal data under real-life conditions and high cost efficiency. Here we present a new method for recording the longitudinal development of tinnitus perception using a modern smartphone application available for iOS and Android devices with no costs for the users. The TrackYourTinnitus (TYT) app is available and maintained since April 2014. A number of 857 volunteers with an average age of 44.1 years participated in the data collection between April 2014 and February 2016. The mean tinnitus distress at the initial measurement was rated on average 13.9 points on the Mini-Tinnitus Questionnaire (Mini-TQ; max. 24 points). Importantly, we could demonstrate that the regular use of the TYT app has no significant negative influence on the perception of the tinnitus loudness nor on the tinnitus distress. The TYT app can therefore be proposed as a safe instrument for the longitudinal assessment of tinnitus perception in the everyday life of the patient.

## Introduction

Tinnitus is the perception of a sound when no corresponding external sound is present. The severity of tinnitus varies largely between tinnitus sufferers. While a large percentage of cases is only minimally impaired during their daily routing, the severe cases of tinnitus are affected by anxiety, depression, insomnia and concentration problems all of which can impair their quality of life (Dobie, 2003; Heller, 2003; Kreuzer et al., 2013). Epidemiological studies of tinnitus indicate a prevalence between 6%–26% with 1.2%–1.6% reporting severely annoying tinnitus (Davis and Rafaie, 2000; Hasson et al., 2010; Gallus et al., 2015). There is currently little evidence for an effective treatment of tinnitus loudness and no pharmacological treatment approved by the US Food and Drug Administration (FDA) or the European Medicines Evaluation Agency (EMEA; Langguth and Elgoyhen, 2012). Among other factors the large heterogeneity of the tinnitus patient population represents a major barrier for the development of effective tinnitus treatments (for a review see e.g., Elgoyhen et al., 2015). A recent review of tinnitus identified at least 13 different types of causal factors for tinnitus (Baguley et al., 2013) that can be described on various dimensions such as its etiology, perceptual characteristics of the sound (i.e., pitch and loudness), time since onset, continuous or intermittent, levels of conscious awareness and perceived distress and comorbidities.

As tinnitus is a purely subjective phenomenon, its assessment is not trivial (Langguth et al., 2007). For the assessment of tinnitus severity standardized questionnaires have been developed, whereas tinnitus loudness can be assessed either by visual analog scale (VAS; Adamchic et al., 2012) or by psychoacoustic measurements. All these measurements are based on the assumption that tinnitus is a rather static phenomenon. However recent data demonstrates that tinnitus loudness and annoyance fluctuate significantly from moment to moment (Henry et al., 2012; Wilson et al., 2015). Henry et al. (2012) conducted a pilot study in which tinnitus symptoms were measured in 24 participants over 2-weeks at four random time points per day using a personal digital assistant (PDA) device. They presented results, which suggest a large between-days variability of tinnitus distress for some study participants. Notably, the frequent measurement of tinnitus over the 2 weeks period had no negative impact on the perceived tinnitus distress as measured by the tinnitus handicap inventory (THI, Newman et al., 1996) indicating that directing the attention on tinnitus several times per day does not worsen the tinnitus (Henry et al., 2012). Using a similar study design (4 assessments per day for 2 weeks), Wilson et al. (2015) also investigated the intra-individual variability of tinnitus distress using smart phone notifications. Likewise, to the study by Henry et al. (2012) they showed that tinnitus fluctuations vary strongly across individuals. Some individuals report strong fluctuations of their tinnitus distress while others report relatively low fluctuations. A coefficient of variation (CV) calculated over the tinnitus self-reports of each participant ranged between 11.5% and 109.9% with a median of 48.4% (Wilson et al., 2015). So far it is not understood why tinnitus fluctuates in some cases and what are the underlying mechanisms for the fluctuations of tinnitus from one moment to the other. Better assessment of the fluctuations of tinnitus is of highest importance for many different reasons: (1) exact assessment of tinnitus fluctuations are of diagnostic importance as intra-individual fluctuations may be an important characteristic feature of an individual’s tinnitus; (2) exact measurement of perceptual fluctuations is a precondition for investigating the neurobiological mechanisms of tinnitus changes over time; (3) knowledge of tinnitus fluctuations over time is important for patient management (e.g., for tinnitus counselling); and (4) information of tinnitus fluctuations is highly relevant for clinical studies, e.g., when effects of specific therapeutic interventions are assessed.

In this article, we want to present a conceptual and technical framework for an ecologically momentary assessment (EMA; Stone and Shiffman, 1994), which allows to systematically assess the moments of different tinnitus symptom severity. The method of EMA (also called Experience sampling method, e.g., Csikszentmihalyi and Larson, 2014) was originally developed for the collection of self-reports of behavior, cognition or emotions in the daily lives of the participants. In the context of tinnitus, we extend this framework to also collect self-reports about the perception of the phantom tinnitus sound and objective measurements of contextual variables (here: sound pressure of the environmental sounds to discover tinnitus masking). The EMA method offers several benefits for the assessment of tinnitus.

First, the EMA approach minimizes the retrospective bias. Several studies from multiple disciplines have demonstrated that multiple biases may jeopardize the retrospective data collection, e.g., in pain reports (Erskine et al., 1990) or for coping strategies (Todd et al., 2004). The retrospective recall is based on a process of mental reconstruction rather than a correct retrieval whereby the current circumstances and the peak of symptoms are emphasized (for a review see e.g., Fredrickson, 2000 on this peak-and-end rule). EMA approaches with momentary assessments of the tinnitus symptoms allow the measurements with exact time stamps in real life and should thereby be well suited for minimizing this retrospective bias.

Second, longitudinal assessment can reveal dynamic processes and temporal relationships. Even though it has been shown that tinnitus symptoms fluctuate from one moment to the other (Henry et al., 2012; Wilson et al., 2015), many questions about this dynamic process are still open: how much varies the extent of fluctuations from one person to the other? How strong are the fluctuations within and between days? Is there a temporal pattern (e.g., tinnitus symptoms are higher/lower in the morning compared to the evening)? Is there a temporal relationship with other factors like stress, emotion, concentration, sleep etc. which can suggest a causal relationship (e.g., emotional arousal increases lead to an increase of tinnitus or vice versa)? Are there context-specific factors for tinnitus improvement or worsening? All these questions can be systematically addressed by the EMA approach at the level of individual subjects (for a review see e.g., Wichers, 2013 about EMA assessment in mental disorders). With this manuscript, we want to introduce the technical platform of the TrackYourTinnitus (TYT) app that will be able to answer a large number of these questions. Some work using TYT has already been published investigating the relationships between tinnitus and emotional states (Probst et al., 2016a) as well as between tinnitus and emotion dynamics (Probst et al., 2016b). Further analyses are currently under preparation.

Third, assessment in real-life situations is characterized by large ecological validity. There are multiple examples in clinical research where a large discrepancy can be observed between the measurements in the clinic (or laboratory) and the measurements in everyday life. For instance, blood pressure readings made by a physician in the clinical context are often higher than the ambulatory blood pressure recordings done outside the clinic—typically described as the “white coat effect” (Hansen et al., 2006). On the other side, measurements of clinical symptoms outside the clinical or laboratory settings are hampered by low controllability, which can lead to noisy signals. An adequate item selection (items should measure the respective moment, should be short and can be answered in a view seconds) with respect to the research question is a key element for using the EMA approach. We consider both types of measurements, in the clinical/laboratory context and in real life, as important for both comprehensive diagnostic assessment and evaluation of treatment effects. While there is already a rich collection of tinnitus research tools than can be used in the laboratory or clinical context, the research tools for assessing tinnitus in real life are still limited and shall be improved by the EMA approach presented here.

Fourth, the technical integration of additional sensor data allows the objective measurement of contextual and biological variables. With the current development, we are using the microphone of the smart phone to measure the environmental sound pressure level for assessing the effect of external sounds on perceived tinnitus loudness and distress. This goes beyond earlier developments where this integration was not possible (Henry et al., 2012; Wilson et al., 2015). In this manuscript we only want to mention this option of the technical platform. Detailed analyses will be reported elsewhere and will also take into account the variance of these measures in the different types of smartphones. Further implementation in the future might also integrate biosensors for objective assessment of biological parameters (e.g., heart rate).

Fifth, the TYT is a non-commercial product and is available all over the world for no costs and without advertisements. To ensure the longitudinal assessment, the technical framework is constantly maintained and updated by the team. Furthermore, the technical implementation allows offline use to ensure that permits the use even in areas without internet connection.

## Materials and Methods

“TYT”^1^ was implemented as a technical realization of the envisaged EMA approach. The TYT platform will be outlined below. A detailed description of the technical aspects is published elsewhere (Pryss et al., 2015a,b; Schickler et al., 2015).

### Recruitment

TYT is an open-access platform at no costs for the users. Upon registration, the users have to agree with the terms of the smartphone app use, which includes that the anonymized data can be used for scientific purposes. The analysis of anonymized data from the smart phone app has been approved by the Ethics Committee of the University Clinic of Regensburg. Before the start of the study, all volunteers agreed with informed consent and no vulnerable populations were involved. To recruit the patients, the TYT app was advertised at the webpages and Facebook pages of the Tinnitus Research Initiative, the TINNET COST Action and the webpage of the participating research groups.

### Data Collection

There are three types of data collected by the TYT platform:

1. The “registration questionnaires” consists of three questionnaires that were completed by the app-user upon registration. The registration questionnaires include the Tinnitus Sample Case History Questionnaire (TSCHQ, Langguth et al., 2007), the short Version of the Tinnitus Questionnaire (miniTQ, Hiller and Goebel, 2004) and a short questionnaire asking for the individually most disturbing tinnitus related aspect.
2. The “state questionnaire” is designed to assess tinnitus and situation-specific variables with eight short questions during everyday life. The state questionnaires constitute the main part of this EMA study. The smartphone app will notify the user at several time points during the day to fill out the state questionnaire. The state questionnaires are delivered randomly within a time frame that can be set by the user (see the “Technical Realization” Section for more details). We decided to give the user more freedom for this setting in order to enhance the usability of the app and allow adaptation to the individual needs. This, however, also enhances the variability in the number of sampling points and the time lag between them. The selection of data analysis methods need to take this into account.
  The state questionnaire consists of eight questions. With the first question we ask the patient if she/he perceives the tinnitus at this moment (answer with yes or no). The second and third question ask about the loudness of the tinnitus and how stressful the tinnitus is. The patients can give their answers on a VAS by moving a slider between the endpoints. Technically, the VAS was implemented as a slider without pre-set values to avoid anchoring affects (Tversky and Kahneman, 1974). The endpoints are “not audible” (question 2) or “not stressful” (question 3) on the left side and “maximal loudness” (question 2) or “maximal stressful” (question 3). The questions 4 and 5 ask for the emotional valence and arousal respectively, using the self-assessment manikins (SAM) developed by Bradley and Lang (1994). Question 6 asks if the person feels currently stressed on a VAS (endpoints “not stressed” and “maximal stressed”). Question 7 asks how much the user concentrated on the task that she/he was doing at the moment (VAS with the endpoints “not at all” and “fully concentrated”). Question 8 asked if the user was irritated at this moment (answer with yes/no).
  The state questionnaire was implemented to allow fast answering by the user. Typically, it takes less than a minute to complete the state questionnaire.
3. The sound pressure of environmental sounds was measured using the built-in microphone of the smartphone while the user was answering the state questionnaire. The microphone recordings were set to record with a resolution of 16 bit for both, the Android and the iOS devices. While inter-subject comparison of the sound pressure measures is limited because of the different smartphone manufacturers, an intra-subject comparison between different moments will be possible. The sound pressure measurements are not reported in this manuscript and is subject to further analyses.

### Technical Realization

**Figure 1:**
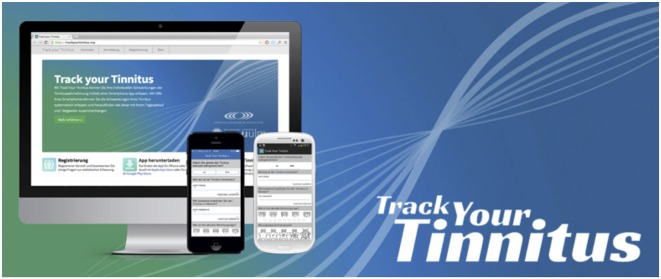
**Frontend of the TrackYourTinnitus (TYT) framework including the website and the smart phone applications for iOS and Android**.

The Front-End (Figure 1) of the TYT includes two smart phone applications for iOS (implemented using Objective C) and Android devices (implemented using Java), and the www.trackyourtinnitus.org website (implemented using the Open-Source PHP-Framework LARAVEL: Code Bright, Otwell, 2016) for the registration and display of the results. Both smart phone apps are freely available for English and German speaking users in the respective app stores. The Back-End consists of a MySQL database running on a Linux Server for the storage of the collected data from all mobile devices. The data on the smart phone devices are stored in the internal SQLite database of the mobile device. If the user is online while he is answering the state questionnaire, the answers between the mobile device and the server will be synchronized immediately. If the user is offline, this synchronization process will be triggered the next time the user is online. In all cases, only anonymized data is transferred and the data transfer is encrypted using the secure sockets layer (SSL) technology. The user has the option to review his own data within the app or download the dataset using the web interface. The anonymized data of all users are stored in a central database maintained by the University of Ulm for research purposes. The user can select between two settings for the state questionnaires: in the “standard settings”, the user receives the state questionnaires at random time points between 8 a.m. and 10 p.m. (can be adjusted) following a randomization algorithm described by Pryss et al. (2015b). A maximum number of 12 state questionnaires per day is allowed by system. In the “custom” settings, the user can define an own schedule with time points where she/he wants to receive the state questionnaire. The TYT platform was developed by the authors of the manuscript (RCP, AB, JS, MR) with additional support by several programmers from the University of Ulm (see “Acknowledgments” Section, Jochen Herrmann, Robin Kraft, Robin Zöller, Aliyar Aras, Marc Schickler). The TYT platform is developed for users with chronic tinnitus. No prior knowledge of EMA is required to use the app.

### Data Management

High emphasis is placed on data management of the TYT platform. Given the automated procedures, the data collection is highly standardized for all participants. Data entry masks are designed carefully to only allow the entry of meaningful data and minimize the risk of wrong entries (e.g., defined value ranges). The data collection of TYT is continuously ongoing. Therefore, strict rules for data analysis were defined in order to reduce reporting biases: at fixed time points during the year, the database is frozen. Data analysis will always be done using the most recent database freeze. The data reported in this article contains the data collected between April 2014 (start of the TYT platform) and February 2016. Further analysis on the dataset have been published elsewhere (Probst et al., 2016a,b) and additional analyses will be performed in the future. This will also include time-series analyses to explore temporal relationships between the tinnitus loudness or distress and the emotional state or the perceived stress level.

### Data Analysis

The influence of the duration of app-use on the perceived tinnitus loudness and distress was tested by means of regression analysis. Statistical analysis was done using the statistical software package R^2^.

## Results

### Study Sample

**Figure 2:**
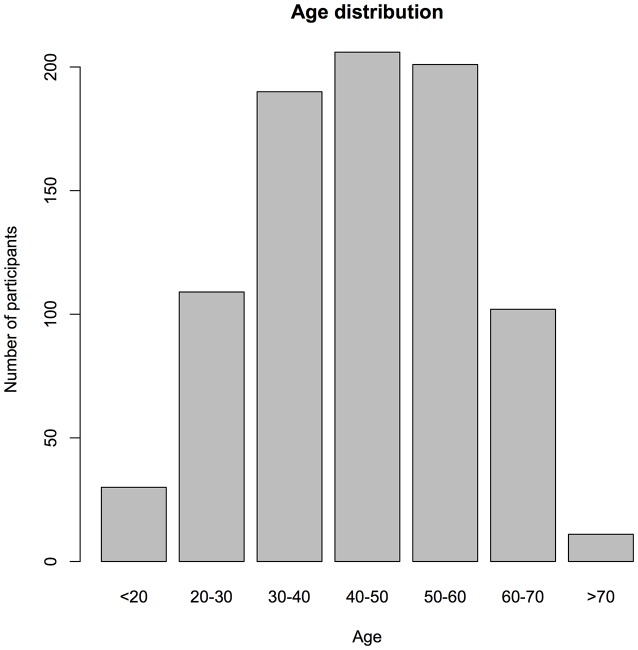
**Age distribution of the participants**.

**Figure 3:**
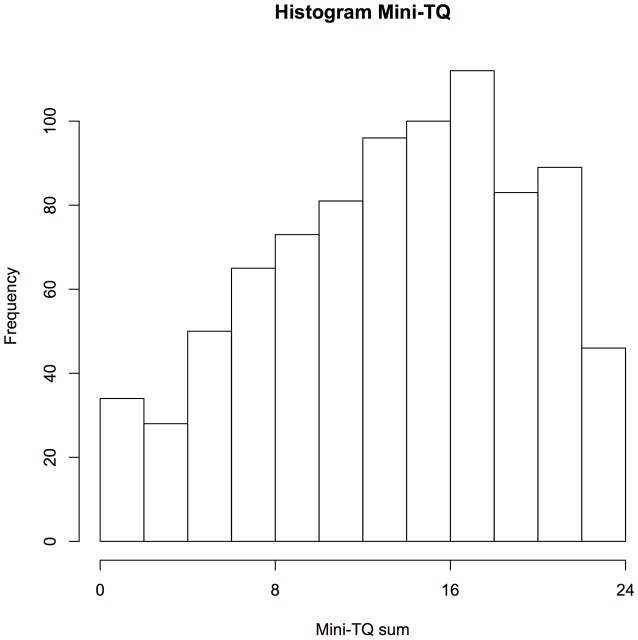
**Histogram of the Mini-Tinnitus Questionnaire (Mini-TQ) sum scores of the participants**. During the registration process, the participants are required to fill out the Mini-TQ. The Mini-TQ measures the strength of the tinnitus distress on a scale from 0 to 24.

A number of 857 volunteers (26.9% female, 73.1% male) participated in the data collection between the launch of the app in April 2014 and February 2016. To recruit the patients, the TYT app was advertised at the webpages and Facebook pages of the Tinnitus Research Initiative, the TINNET COST Action and the webpage of the participating research groups. The average age of the participants was 44.1 years (standard deviation: 14.1 years, Figure 2). Upon registration, the participants completed the Mini-Tinnitus Questionnaire (Mini-TQ) for the assessment of tinnitus-related distress. The average sum score over all participants was 13.9 points (SD 6.0, Figure 3, maximum score of the Mini-TQ is 24 points).

### Influence of Study Participation on Tinnitus Symptoms

An important question is whether the continuous participation in the study, with repeated measurements of tinnitus-related symptoms, leads to a worsening or improvement of tinnitus symptoms. To analyze this question, we selected a subgroup of subjects that have used the app regularly for at least a month. Linear regression analysis was calculated for these users (*n* = 66) in this timeframe to test the influence of the study duration on the tinnitus loudness.

The duration of app-use measured in days was used as a regressor in this model, the tinnitus loudness as regressand (tinnitus loudness ~ app-use duration). Duration of app-use did not predict tinnitus loudness, *β* < 0.001, *t*_(6291)_ = 1.26, *p* = 0.21. Furthermore, the duration of the app-use did not explain the variance of the tinnitus loudness significantly, R-squared <0.001, *F*_(1,6291)_ = 1.58, *p* = 0.21. Similarly, we investigated the influence of the duration of app-use on tinnitus distress (tinnitus distress ~ app-use duration). The duration of app-use did not predict tinnitus distress, *β* < 0.001, *t*_(6264)_ = 1.3, *p* = 0.19. Also, the duration of the app-use did not explain the variance of the tinnitus distress significantly, R-squared <0.001, *F*_(1,6264)_ = 1.7, *p* = 0.19. Additionally, we calculated a paired *t*-test comparing the mean of the first 5 sampling state questionnaires against the mean of the last 5 state questionnaires for each user. Again, there was no significant influence of time neither on tinnitus loudness (*t*_(61)_ = 1.25, *p* > 0.2) nor on tinnitus distress (*t*_(61)_ = 0.38, *p* > 0.7), indicating, that app-use did not have a negative impact on the users’ tinnitus.

The same type of paired *t*-test was repeated for the subjects, which used the app for less than 1 month. We selected all users with more than 5 days of app-use and less than 30 days of use. Also for this group of users, there was no significant influence of time neither on tinnitus loudness (*t*_(133)_ = 0.53, *p* > 0.5) nor on tinnitus distress (*t*_(129)_ = 0.79, *p* > 0.4).

**Figure 4:**
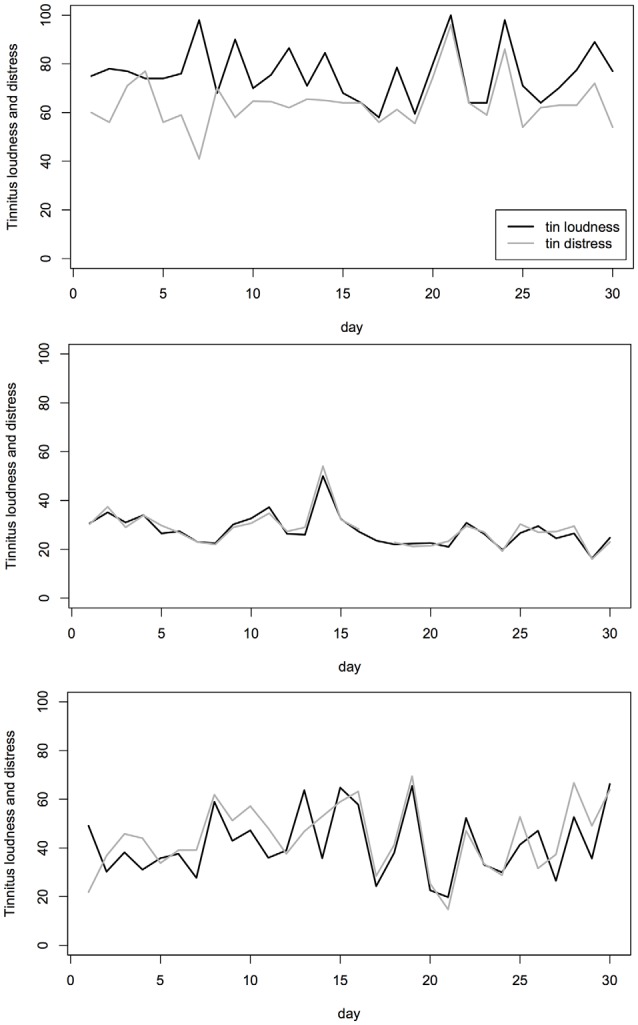
**Example of three individual tinnitus patients**. Measurements of the tinnitus loudness (black) and tinnitus distress (gray) on 30 consecutive days are shown for the three individuals. The tinnitus loudness and tinnitus distress were both measured on a visual analog scale (VAS) and converted to numerical values in the range of 0 (“not audible”/“not stressful”) to 100 (“maximal loudness”/“maximal stressful”).

To give an impression of the fluctuation of tinnitus loudness and tinnitus distress over time, a random selection of three participants is illustrated in Figure 4.

## Discussion

We have introduced the TYT app as a new platform that allows the assessment of tinnitus-related symptoms during the everyday life of people with tinnitus. The app is available in the two major app stores for Android and iOS devices. Between April 2014 and February 2016, the app was used by 857 people with tinnitus, with the majority of users between 30 and 60 years old. The amount of tinnitus-related distress as measured with the Mini-TQ covered the full range of the measurement spectrum showing that the app was used by people with various levels of tinnitus distress. However, further studies will need to clarify, whether the population using the app is representative to the general tinnitus population. The recruitment of patients via the internet and the app stores introduces a new way in patient recruitment compared to earlier studies. It is therefore important to mention that our results are in line with the previous studies on mobile assessment of tinnitus (Henry et al., 2012; Wilson et al., 2015). The large amount of sampled data allows the analysis of more specific research questions about tinnitus which are reported elsewhere (Probst et al., 2016a,b).

From a clinical point of view, there could be the concern that the tinnitus increases by the repeated assessment (and therefore a reminder) of the tinnitus perception. It is notable here that the tinnitus perception did not change as a result of repeated sampling, neither tinnitus loudness nor tinnitus distress. This is in line with the study by Henry et al. (2012) in which it was shown that tinnitus did not change during a 2 week assessment period with four measurements each day. Furthermore, we also analyzed the data of users with only a short duration of app-use. In principle it would be possible that users where the app-use has a negative impact on their tinnitus stop using the app, while the users without negative impact use the app for a longer time. This is clearly not the case since the paired *t*-tests on the subgroup of subjects with short app-use duration did not reveal a significant difference between the first and the last state measurements either. These findings further support the notion that assessment of tinnitus symptoms during everyday life is a safe method that is not increasing neither tinnitus loudness nor tinnitus distress.

The aim of the TYT platform is: (1) to enable real-time assessment of tinnitus variations and reduce the effect of recall bias; (2) to investigate the factors influencing the increase and decrease of tinnitus symptoms at the individual subject level; (3) to enable the longitudinal assessment of tinnitus symptoms and their dynamic processes; (4) to introduce tinnitus measurements with higher ecological validity in real-life conditions as opposed to tinnitus measurements in the lab or the clinic, and therefore establish ecological momentary assessment (EMA) methods for tinnitus research; and (5) to implement and validate tinnitus assessment with smart phones as a cost-effective assessment tool that can be applied for large populations.

Future technical implementation will also enable monitoring of clinical treatments. The effects of clinical treatments are currently typically assessed by weekly or monthly questionnaires and can therefore hardly be used to investigate dynamic changes during the treatment phase. Although EMA has been used in other domains to monitor treatment progress, e.g., for antidepressant treatment (Barge-Schaapveld and Nicolson, 2002), it has not been used for the assessment of tinnitus treatments yet. Another benefit of EMA was revealed by the study of Barge-Schaapveld and Nicolson (2002), which demonstrated an increased reporting of side-effects. The side effect of increased dizziness was reported by 35 patients using EMA while only seven patients reported increased dizziness to their general practitioner (Barge-Schaapveld and Nicolson, 2002). This suggests that the recall bias of retrospective assessment not only influences the measurement of treatment related symptom reduction but also the reporting of treatment-related side effects. Additionally, frequent assessment of treatment effects with smart phones could help to learn more about reasons for study drop out by providing more precise data of what has happened immediately before drop out.

However, beside all the excitement about the new opportunities and advantages of EMA, we also want to mention the drawbacks of this method, which mainly arise from the low controllability of our sampling method. Since the assessment with smart phones is not completed under the supervision of clinical staff, the correct usage of the device as well as the identity of the user cannot be guaranteed. Also, there is little control over the situation and circumstances of the app usage, which on one side enhances the ecological validity of the measurement, but on the other side reduces its controllability. It is, therefore, important to mention that EMA should not be considered as a substitute for standard questionnaires used in the clinical routine, but rather be seen as a complementary method with additional value for both clinical practice and for basic as well as clinical research. We are looking forward to a new wave of studies using EMA for its various applications in tinnitus research.

## Author Contributions

WS: substantial contribution to the design of the study, data analysis, conception and implementation of the TrackYourTinnitus app, drafted and revised the manuscript. RCP: substantial contribution to the design of the study, data analysis, conception, implementation and maintenance of the TrackYourTinnitus app, drafted and revised the manuscript. TP and BL: substantial contribution to the design of the study and data analysis, drafted and revised the manuscript. JS, AB and MR: substantial contribution to the conception, implementation and maintenance of the TrackYourTinnitus app.

## Conflict of Interest Statement

The authors declare that the research was conducted in the absence of any commercial or financial relationships that could be construed as a potential conflict of interest.

## Footnotes

^1^www.trackyourtinnitus.org

^2^www.r-project.org, version 3.3.1.
